# Designed proteinoid polymers and nanoparticles encapsulating risperidone for enhanced antipsychotic activity

**DOI:** 10.1186/s12951-020-00709-z

**Published:** 2020-10-21

**Authors:** L. Lugasi, I. Grinberg, S. Rudnick-Glick, E. Okun, H. Einat, S. Margel

**Affiliations:** 1 Department of Chemistry, The Institute of Nanotechnology and Advanced Materials, Bar-Ilan University, 5290002 Ramat Gan, Israel; 2 The Mina and Everard Goodman, Faculty of Life Sciences, Bar-Ilan University, 5290002 Ramat Gan, Israel; 3 The School of Behavioral Sciences, Tel Aviv-Yaffo Academic College, 6818211 Tel Aviv, Israel

**Keywords:** Proteinoid nanoparticles, Self-assembly, Risperidone, Drug delivery, Antipsychotic therapy

## Abstract

**Background:**

Nanoparticles (NPs) incorporating drug formulations can be used to facilitate passage through biological barriers including the blood–brain barrier (BBB) and increase drug delivery and bioavailability. Hence, NP-based administration may enhance the efficiency of current antipsychotics. Encapsulation within NPs can resolve aqueous solubility problems that not only reduce permeability through the BBB but also affect targeting. The present study describes a new drug delivery system based on proteinoid NPs to explore the possibility of improving drug efficacy. Risperidone (RSP) is a commonly used atypical antipsychotic medication, and was therefore selected for encapsulation by proteinoid NPs.

**Results:**

Proteinoid polymers with high molecular weight and low polydispersity were synthesized from l-amino acids and poly-l-lactic acid (PLLA) by thermal step-growth polymerization mechanism. RSP-loaded proteinoid NPs were then prepared using a self-assembly process in the presence of RSP, followed by PEGylation. The optimal PEGylated RSP-loaded NPs were characterized in terms of diameter and size distribution, drug loading, ζ-potential, cytotoxicity, biodistribution, and psychopharmacological effects. The findings indicate significantly higher antipsychotic activity of drug-loaded proteinoid NPs compared to free RSP.

**Conclusions:**

Proteinoid NPs enhance RSP delivery and may potentially increase drug efficiency by reducing dosage and side effects.
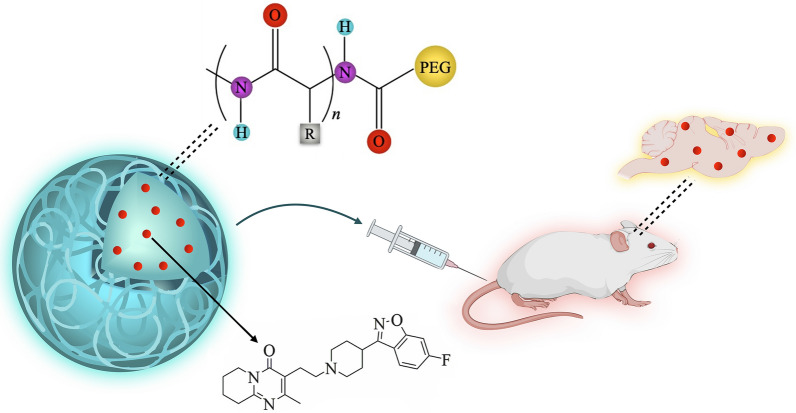

## Background

Mental disorders are cognitive behavioral or emotional patterns that cause significant distress and/or permanent impairment of personal functions [1]. They affect approximately 13% of the global population. Signs and symptoms vary, ranging from mild to moderate and even severe impairment. Triggers of mental disorders include genetics [2], biochemical processes in brain structure and development [3], environmental changes [4], traumatic life events [5], drug use, and lifestyle [6]. Treatment is disorder- and individual-specific and usually combines psychotherapy and medication.

**Fig. 1:**
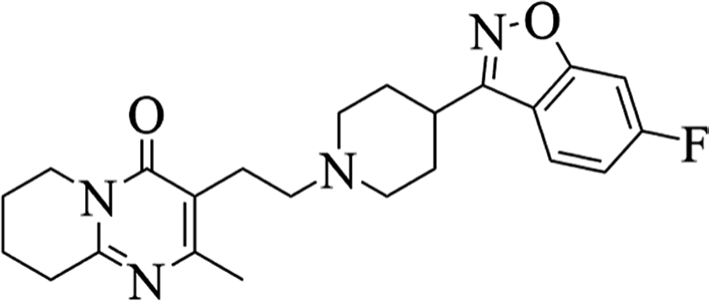
Chemical structure of risperidone (RSP)

Antipsychotics (also called neuroleptics or major tranquilizers) are a class of medications that are primarily used in the treatment of psychoses, in particular schizophrenia and bipolar disorders [7]. Risperidone (RSP, Fig. 1) is considered as one of the most reliable and effective atypical (second generation) antipsychotic medications, and is widely used in the treatment of irritability associated with autism and both positive (e.g., hallucinations or delusions) and negative (e.g., emotional withdrawal or loss of speech) symptoms of schizophrenia and bipolar disorders [8–10]. Schizophrenia and bipolar disorders are progressive illnesses with an incidence of approximately 1–2.5% of the population.

Risperidone, 3-{2-[4-(6-fluoro-1,2-benzisoxazol-3-yl)-1-pip-eridin]ethyl}-6,7,8,9-tetrahydro-2-methyl-4H-pyrido-[1,2-a]pyrimidine-4-one, is a benzoisoxazole derivative with strong binding affinity for serotonin 5-HT2 and dopamine D2 receptors, as well as high affinity to α_1_- and α_2_-adrenergic receptors and histamine H_1_ receptors [11, 12]. In humans, RSP undergoes extensive metabolism mainly by hydroxylation and oxidative *N*-dealkylation to a 9-hydroxyrisperidone metabolite that is subjected to generic polymorphism [13]. Recent studies have shown that polymorphism of the CYP2D6 gene plays an important role in RSP metabolism by the enzyme expressed in the blood, and indicate that RSP is a highly unstable molecule and a poorly water-soluble weak base, which belongs to Class II of the Biopharmaceutics Classification System. This leads to erratic bioavailability requiring frequent dose revision [14].

Atypical antipsychotics are less likely to cause extrapyramidal side effects. Although RSP is less sedating than other atypical antipsychotics, it tends to have sexual side effects and after-effects of increasing the risk of weight gain and metabolic problems, and is likely to have more extrapyramidal side effects [12, 15]. Therefore, there is a need to find effective formulations of these hydrophobic therapeutic compounds to improve solubility, stability and drug efficiency and reduce side effects.

Biodegradable NPs have gained importance in the field of nanomedicine as they are able to improve targeted drug delivery, biocompatibility, bioavailability, safety, permeability and retention time and reduce toxicity [16–21]. Formulation-based biodegradable NPs are efficient vehicles for drug transport to targeted sites. Previous studies demonstrated that precisely designed biodegradable NP carriers can cross the blood brain barrier (BBB), improve administration, and control drug targeting [20–24].

Proteinoids are random polymers consisting of amino acids, synthesized by thermal step-growth polymerization [25–30]. These polymers are biodegradable and resemble natural proteins, thus expressing non-immunogenic and non-toxic characteristics [31–33]. One notable advantage of proteinoids as synthetic polymers is that they can be specifically designed to suit a wide range of properties in biomedical applications, including drug delivery systems [34].

In an aqueous solution, by means of a self-assembly mechanism, proteinoids can fold to form hollow particles [35]. The self-assembly process is made possible by the presence of the many functional groups that form part of the random polymer backbone. In an aqueous solution, the hydrophobic residues of the proteinoid are assembled to form a hydrophobic core within the particle matrix to minimize their contact with the aqueous continuous phase, while the hydrophilic residues are exposed to form hydrogen bonds on the particle surface [36, 37]. The hollow interior of the particles, which is formed during self-assembly, can be utilized to encapsulate various molecules, making proteinoids NPs biocompatible drug carriers [38]. Earlier studies by our group demonstrated that encapsulation of therapeutics such as anti-cancer drugs and near infrared (NIR) fluorescent dyes have potential use in cancer diagnosis and therapy [32, 39, 40].

In the present study, a series of proteinoids with high molecular weight and low polydispersity were designed from l-amino acids in the presence and absence of poly(l-lactic acid) (PLLA). PLLA was incorporated within the copolymer backbone to enhance the biodegradability and elicit mechanical rigidity [41–43].

High drug loading (DL) capacity and prolonged blood stream circulation are essential requirements for drug carriers [44]. Polyethylene glycol (PEG) coating (PEGylation) shields the NP surface from aggregation and phagocytosis and thereby impacts NPs administration; it prolongs the retention time in the blood stream, improves delivery of the therapeutic cargo, enhances passage through the BBB, and facilitates distribution and clearance from the body [45–49].

Encapsulation of RSP in nano-carrier delivery systems such as biodegradable NPs was hypothesized to stabilize it; along with drug dosage tailoring, such encapsulation may enhance the tolerability and adherence and increase the bioavailability, thereby improving the antipsychotic activity and reducing the side effects. For this purpose, a psychostimulant-induced behavioral model, which is frequently used to model facets of schizophrenia and mania, especially in the context of screening potential novel treatments, was carried out. Whereas a number of psychostimulants are used in this context, the more established drug is amphetamine [50]. Specifically, in the amphetamine-induced hyperactivity model, single or multiple doses of amphetamine are administered to a model animal at appropriate doses that induce hyperactivity (including increased movement during a testing session) as well as other behavioral changes. Antipsychotic drugs, including RSP [8] and other mood stabilizers [51] were repeatedly demonstrated to ameliorate amphetamine-induced behavioral changes. Accordingly, this model is accepted as a screening model for antipsychotic effects [52]. Here, several PEGylated RSP-loaded proteinoid NPs (Prot./RSP) were introduced in order to increase the stability and solubility of the drug in the aqueous continuous phase, as well as to improve targeting to the brain.

## Results

### Design and characterization of proteinoids

Four different proteinoid polymers were synthesized from Lys or Glu, together with Phe and His, in absence or presence of PLLA, as described in the methods section (see Table 4). The molecular weight (Mw) and polydispersity index (PDI) were determined by gel permeation chromatography (GPC, see Table 1). The basic proteinoids, Prot.1 and Prot.3, exhibited Mw of 168.0 and 154.2 kDa, respectively, with similar PDI of 1.00, whereas the acidic proteinoids, Prot.2 and Prot.4 had Mw of 142.6 and 132.3 kDa with PDI of 1.02 and 1.05, respectively. Prot.1–4 exhibited optical activity of − 11.6, − 6.5, 7.8 and 4.8, respectively.

**Table 1:** Mw, Mn, Mp, PDI and optical activity of the proteinoids

Proteinoid^a^	Mw^b^ (kDa)	Mn^b^ (kDa)	Mp^b^ (kDa)	PDI^c^ (kDa)	Optical activity [a]_D_^25 °C^ (°)^d^
Prot.1	168.0	166.3	156.8	1.00	− 11.6
Prot.2	142.6	139.5	135.1	1.02	− 6.5
Prot.3	154.2	153.9	149.3	1.00	7.8
Prot.4	132.3	126.0	126.4	1.05	4.8

^a^The basic Prot.1,3 and acidic Prot.2,4 proteinoids were prepared at 140 and 180 °C, respectively

^b^Molecular weights were measured by GPC, Mp is the molecular mass at the peak

^c^PDI is the polydispersity index, given by Mw/Mn

^d^Specific optical rotation (c = 1 in H_2_O at 25 °C)

### Formation of hollow and RSP-loaded proteinoid NPs

Proteinoids were self-assembled to form proteinoid NPs, followed by PEGylation process. The PEGylated hollow NPs (Prot.1–4) in water as a continuous phase had diameters of less than 95 nm with a narrow size distribution (SD) of 2–7%, in accord with the low PDI of the proteinoid polymers. The hollow NPs, Prot.3 and Prot.4, prepared in the absence of PLLA, yielded diameters of 94 ± 7 and 90 ± 4 nm, respectively, while Prot.1 and Prot.2 that incorporate PLLA were smaller with diameters of 78 ± 4 and 58 ± 2 nm, respectively. The RSP-loaded NPs (Prot.1–4/RSP NPs) with 20% w/w RSP had a slightly higher diameter range of 86 ± 3 to 122 ± 6 nm, with a similar narrow SD of 2–6%.

**Fig. 2:**
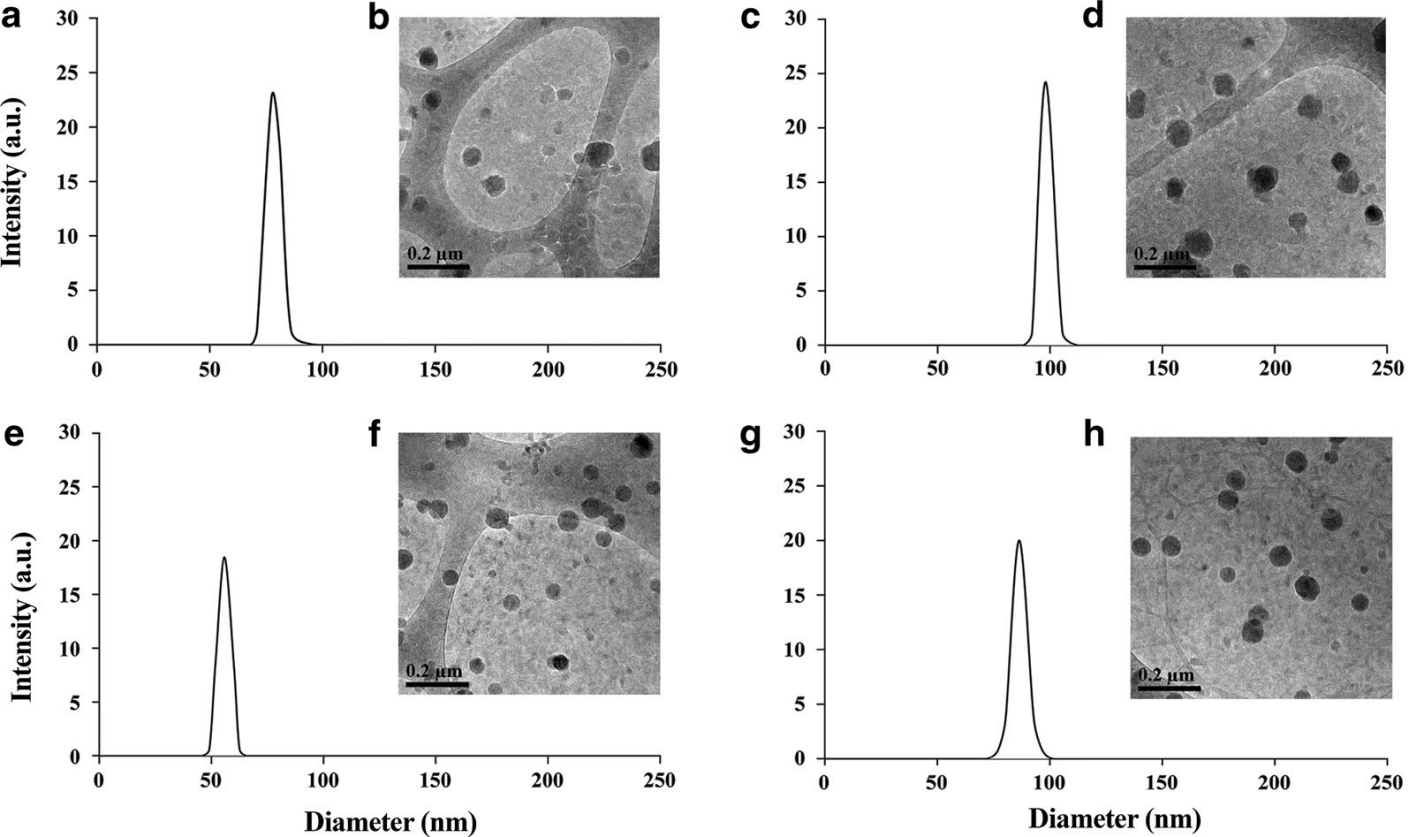
Diameter histograms by DLS and cryo-TEM images of Prot. 1 (**a**, **b**), Prot. 1/RSP (**c**, **d**), Prot. 2 (**e**, **f**) and Prot. 2/RSP (**g**, **h**) NPs dispersed in aqueous continuous phase

**Fig. 3:**
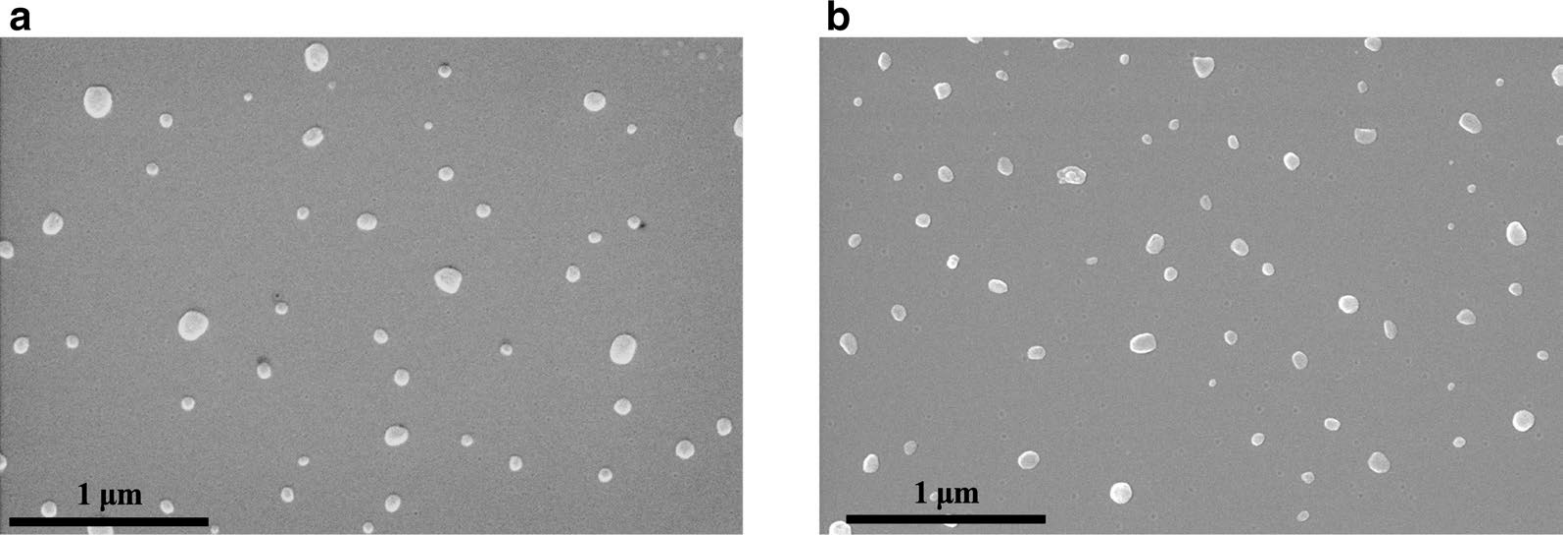
SEM images of Prot.1/RSP (**a**) and Prot.2/RSP (**b**) NPs

The hydrodynamic diameters of the hollow Prot.1 and Prot.2 and their corresponding RSP-loaded NPs, Prot.1/RSP and Prot.2/RSP, dispersed in aqueous continuous phase were analyzed by dynamic light scattering (DLS) and cryogenic transmission electron microscopy (cryo-TEM), as shown in Fig. 2 and Table 2. Prot.1 exhibited a population of uniform spherical hollow NPs with diameter of 78 ± 4 nm by DLS as shown in the histogram (Fig. 2a and Table 2) and similar diameter of 75 ± 8 nm by cryo-TEM (Fig. 2b). Prot.1/RSP produced uniform spherical NPs with higher diameter of 97 ± 2 nm by DLS (Fig. 2c and Table 2) and 93 ± 6 nm by cryo-TEM (Fig. 2d). Prot.2 was smaller than Prot.1 and yielded a population of uniform spherical hollow NPs with a diameter of 58 ± 2 nm by DLS (Fig. 2e and Table 2) and 58 ± 3 nm by cryo-TEM (Fig. 2f). Prot.2/RSP also presented smaller diameter compared to Prot.1/RSP of 86 ± 3 nm (DLS, Fig. 2g and Table 2) and 82 ± 2 nm (cryo-TEM, Fig. 2h). These results show that both methods for measuring NP diameter provide similar results; commonly, the diameters measured from the cryo-TEM were the same or slightly lower (by up to 5%) than those obtained by the DLS. The dry diameters of Prot.1/RSP and Prot.2/RSP were measured by scanning electron microscopy (SEM, Fig. 3), yielding 74 ± 4 and 55 ± 5 nm, respectively.

**Table 2:** Hydrodynamic diameter and SD, ζ-potential and DL of PEGylated proteinoid NPs

Proteinoid NPs	Diameter (nm)	ζ-potential (mV)	DL (w/w%)
Prot.1	78 ± 4	12 ± 3	–
Prot.2	58 ± 2	− 10 ± 4	–
Prot.3	94 ± 7	12 ± 1	–
Prot.4	90 ± 4	− 8 ± 2	–
Prot.1/RSP	97 ± 2	13 ± 3	20 ± 0.1
Prot.2/RSP	86 ± 3	− 16 ± 1	20 ± 0.1
Prot.3/RSP	120 ± 5	13 ± 3	19 ± 0.4
Prot.4/RSP	122 ± 6	− 12 ± 2	19 ± 0.3

Proteinoid particles were formed by a self-assembly process in aqueous 10 µM NaCl solution containing 1% DMSO. The initial RSP concentration was 20% w/w relative to the proteinoid. The hollow proteinoids and Prot./RSP NPs were PEGylated. The hydrodynamic diameter was measured by DLS, the ζ-potential was measured at pH = 7.4 by a ζ-potential analyzer, and the DL was measured by HPLC using a calibration curve of standard RSP solutions

The ζ-potentials at pH = 7.4 (Table 3) were 12 ± 3 and 12 ± 1 mV for the basic hollow proteinoids NPs, Prot.1 and Prot.3, respectively, while the corresponding values for the acidic proteinoids NPs were slightly lower, − 10 ± 4 and − 8 ± 2 mV for Prot.2 and Prot.4, respectively. The RSP-loaded NPs (Prot.1–4/RSP) exhibited higher negative or positive ζ-potential values of 13 ± 3, − 15 ± 1, 13 ± 3 and − 12 ± 2 mV.

**Table 3:** Hydrodynamic diameter and SD, ζ-potential and DL of Prot.1,2/RSP NPs loaded with various concentrations of RSP

Prot./RSP (%)	Diameter (nm)	ζ-potential (mV)	DL (w/w%)
Prot.1/RSP (10%)	68 ± 2	12 ± 1	10 ± 0.1
Prot.1/RSP (20%)	97 ± 4	13 ± 3	20 ± 0.1
Prot.1/RSP (30%)	143 ± 5	17 ± 2	30 ± 0.1
Prot.1/RSP (40%)	178 ± 8	18 ± 2	40 ± 0.3
Prot.1/RSP (50%)	215 ± 7	19 ± 3	50 ± 0.2
Prot.2/RSP (10%)	68 ± 2	− 10 ± 2	10 ± 0.1
Prot.2/RSP (20%)	86 ± 3	− 16 ± 1	20 ± 0.1
Prot.2/RSP (30%)	136 ± 5	− 16 ± 2	30 ± 0.3
Prot.2/RSP (40%)	156 ± 8	− 17 ± 1	40 ± 0.2
Prot.2/RSP (50%)	194 ± 9	− 19 ± 3	50 ± 0.4

Proteinoid particles were formed by a self-assembly process in 10 µM NaCl aqueous solution containing 1% DMSO. The initial RSP concentration was between 10 and 50% w/w relative to the proteinoid. The hydrodynamic diameter was measured by DLS, the ζ-potential was measured at pH = 7.4 by a ζ-potential analyzer, and the DL was measured by HPLC using a calibration curve with standard RSP solutions

The DL capacity of Prot.1–4 was determined by high performance liquid chromatography (HPLC) using standard calibration solutions of RSP [7]. The drug was dissolved in DMSO (1% relative to the total aqueous dispersion volume) to 20% w/w compared to the total proteinoid weight. Table 2 presents the DL (w/w %) of Prot. 1–4/RSP NPs, 20 ± 0.1, 20 ± 0.1, 19 ± 0.4 and 19 ± 0.3%, respectively. As shown in the table, Prot.1 and Prot.2 offer better RSP encapsulation owing to the significantly smaller diameters of 78 ± 4 and 58 ± 2 nm, respectively (compared to 94 ± 7 and 90 ± 4 nm for Prot.3 and Prot.4, respectively). Prot. 2 also has greater physical stability as indicated by the higher ζ-potential of − 10 ± 4, compared to − 8 ± 2 mV for Prot.4. In addition, Prot.1 and Prot.2 also show slightly higher RSP DL of 20 ± 0.1% w/w compared to 19 ± 0.3/0.4% w/w for Prot.3,4.

Table 3 presents an optimization study of PEGylated Prot.1/RSP and Prot.2/RSP NPs. Encapsulation of RSP at 10, 20, 30, 40 and 50% w/w within Prot.1 formed larger particle diameters of 68 ± 2, 97 ± 4, 143 ± 5, 178 ± 8 and 215 ± 7 nm compared with 68 ± 2, 86 ± 3, 136 ± 5, 156 ± 8 and 194 ± 9 nm for Prot.2. These results indicate that 20% RSP yields an optimal diameter of 68 ± 2 for both Prot.1/RSP and Prot.2/RSP NPs. Higher RSP concentrations yield particles with diameters above 100 nm, which are less suitable for drug delivery.

### In vitro cell viability assay

**Fig. 4:**
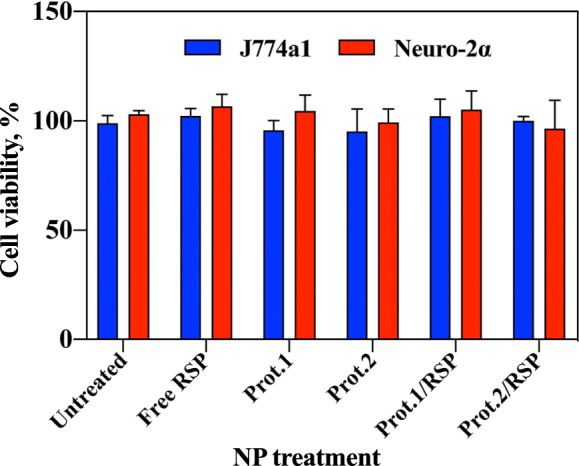
Cellular mitochondrial metabolic rate by XTT assay in J774A.1 and Neuro-2α cell lines following 48 h exposure to Prot.1, Prot.2, Prot.1/RSP and Prot.2/RSP PEGylated proteinoid NPs with 10% PLLA

XTT-based viability assays in cell culture are used to study changes in the number of cells and their metabolic activity. The assay is based on extracellular reduction of XTT by NADH produced in the mitochondria by trans-plasma membrane electron transport and an electron mediator. The effect of PEGylated hollow and RSP-loaded proteinoid NPs on J774A.1 and Neuro-2α cells was examined by XTT (Fig. 4). Prot. 1 and Prot.2 as well as Prot1/RSP and Prot.2/RSP NPs are not toxic to both cells, showing viability levels close to 100%, similar to the control group (free RSP).

### In vitro cell uptake study

**Fig. 5:**
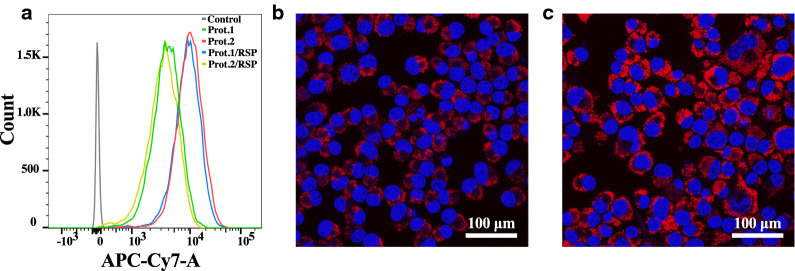
Intracellular fluorescence of Cy7-conjugated Prot.1 (green), Prot.2 (yellow), Prot.1/RSP (blue) and Prot.2/RSP (red) NPs of Neuro-2α cells by FACS (**a**). Fluorescence microscopy images of cellular uptake of Cy7-conjugated (cell membrane, red) of Prot.1 (**b**) and Prot.2 (**c**) and Hoechst dye (cell nucleus, blue) in Neuro-2α following 24 h exposure

Cell permeability and fluorescent microscopy images of Neuro-2α cell of Cy7-cojugated PEGylated hollow and RSP-loaded Prot.1 and Prot.2 were analyzed. The study was carried out using flow cytometry and visualized by confocal microscope. Cell nuclei were stained with Hoechst 33342 [53]. Figure 5 exhibits the intracellular fluorescence of Prot.1, Prot.2, Prot.1/RSP and Prot.2/RSP. Figure 5a demonstrates fluorescence-activated cell sorting (FACS) analysis of murine Neuro-2α cell uptake after 4 h of all four Cy-7 conjugated NPs. Figure 5b and c present the intracellular fluorescence of the NPs on Neuro-2α and show that the NPs penetrate the membrane and are taken up by the cells.

### In vivo biodistribution studies

**Fig. 6:**
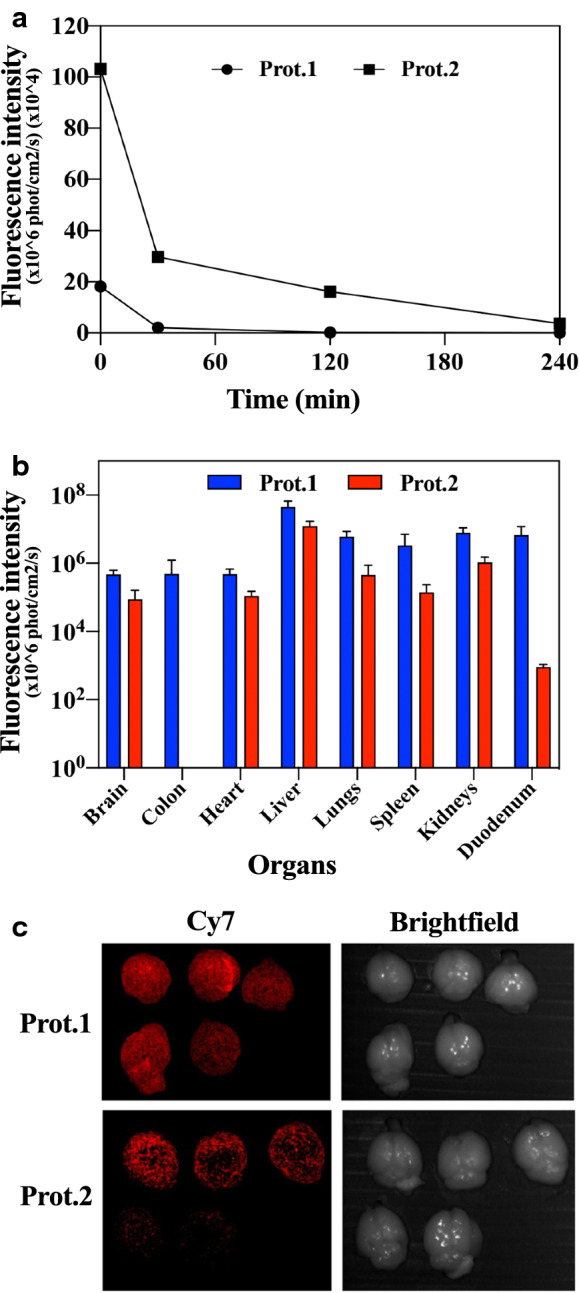
Biodistribution of proteinoid NPs. (**a**) Blood clearance of Prot.1,2 NPs. 100 μL of Cy7-conjugated Prot.1 and Prot.2 NPs (0.2 mg/mL) were IV injected via the tail vein and blood was drawn at different time intervals, (**b**) Fluorescence of Cy7-conjugated Prot.1,2 NPs in different organs. Mice were sacrificed 4 h post injection and organs were harvested. (**c**) Fluorescent signals and brightfield images of Cy7-conjugated Prot.1,2 NPs using the Maestro in vivo imaging system

In order to determine the in vivo body distribution and blood half-life of Cy7-conjugated Prot.1 and Prot.2, 0.2 mg/mL of each proteinoid NP were injected IV into the tail vein of male BALB/C mice. Blood was drawn at 0, 30 min, 1 h and 4 h post injection. The fluorescence decreased over time, and only traces of fluorescence were noticed 4 h post injection. The blood half-lives of the Cy7-conjugated proteinoid NPs were calculated to be approximately 20 min (Fig. 6a). Four hours post injection organs were harvested, and a fluorescent signal was obtained from the brain, colon, heart, lungs, spleen, kidneys and duodenum (Fig. 6b).

### In vivo psychopharmacological effect

The amphetamine-induced hyperactivity screening model was used to evaluate the in vivo psychopharmacological effects of RSP-loaded proteinoid NPs. These NPs (Prot.1,2/RSP 20%) were injected IV immediately before the testing session; saline, free RSP, and hollow NPs served as controls. Exploration-related parameters including distance travelled, time mobile and center time in an open field arena were digitally recorded and analyzed using the ANY-maze software.

**Fig. 7:**
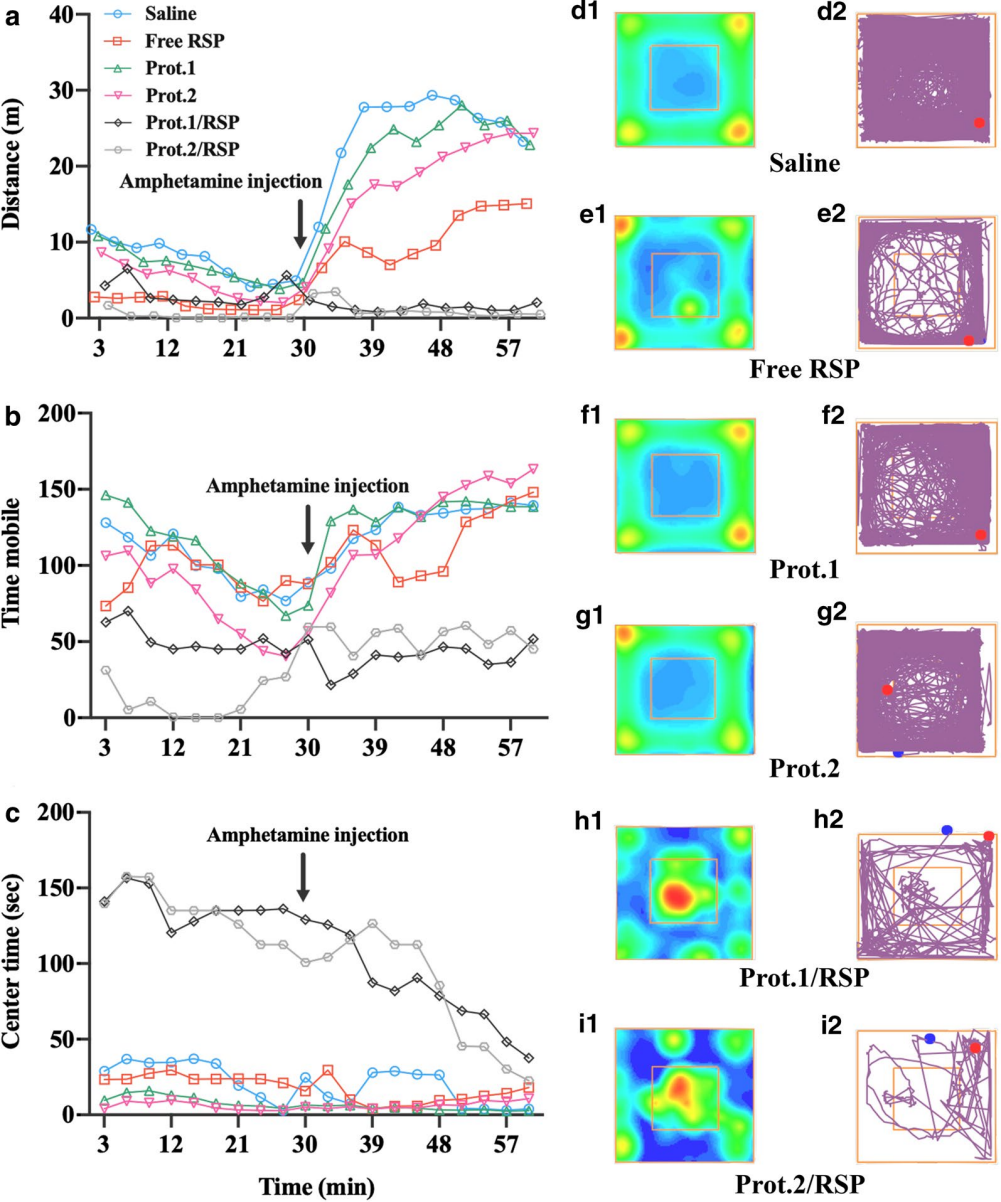
Analysis of open field test NPs treatment prior (0–30 min) and post (30–60 min) amphetamine IP uptake using ANOVA. Recorded tracking of amphetamine-induced hyperactivity screening model during 60 min session of distance travelled (**a**), time mobile (**b**) and center time (**c**). Occupancy heat maps and plots produced by the ANY-maze software (**d**_1-2_–**i**_1-2_)

As expected, the administration of amphetamine resulted in increased distance travelled, and RSP reduced distance travelled during the session as shown in Fig. 7a. Analysis of variance (ANOVA) indicated significant effect of amphetamine to increase distance [F(1, 41) = 202.1, p < 0.001], effect of treatment to reduce distance [F(5, 41) = 29.3, p < 0.001], and interaction [F(5, 41) = 20.3, p < 0.001]. Post-hoc analysis of the first 30 min of the session (before amphetamine administration) demonstrated that all RSP groups had lower distance compared with saline control (p < 0.001; Cohen’s d > 2.5). Following amphetamine administration, all RSP groups exhibited reduced activity compared with saline control (p < 0.001; Cohen’s d > 2.12). Prot.2/RSP treatment had a stronger effect compared with free RSP (p = 0.043; Cohen’s d = 1.88) with a similar trend for Prot.1/RSP (p = 0.09; Cohen’s d = 1.73).

In addition, amphetamine treatment significantly increased time mobile and RSP treatment significantly decreased time mobile (Fig. 7b). A significant effect of amphetamine [F(1, 41) = 13.95, p = 0.001] and RSP treatments [F(5, 41) = 10.82, p < 0.001] without interaction [F(5, 41) = 1.36, p = 0,26] were observed using ANOVA. Post-hoc analysis of the first 30 min of the session demonstrated that mice treated with Prot.1,2/RSP had lower time mobile compared with saline control group (Prot.1,2/RSP vs. saline—p ≤ 0.003, Cohen’s d ≥ 1.53). Post-hoc analysis for the second half of the session (after amphetamine administration) showed that treatment with RSP-loaded proteinoid NPs (but not free RSP) reduced time mobile compared with saline control (p ≤ 0.03). In addition, the effects of RSP-loaded proteinoids had a trend to reduce time mobile compared with animals treated with free RSP (Prot.1/RSP *vs.* free RSP—p = 0.055, Cohen’s d = 1.66 and Prot.2/RSP vs. free RSP—p = 0.1, Cohen’s d = 1.43).

Time in the center of the open field is considered a measure of anxiety-like behavior [51]. As shown in Fig. 7c, amphetamine administration resulted in decreased time in the center of the arena [F(1, 41) = 17.18, p < 0.001]. In contrast, a potent effect to increase time in the center was shown in animals treated with RSP-loaded proteinoid NPs [(F(5, 41) = 15.48, p < 0.001], and there was a significant interaction between the two factors [F(5, 41) = 3.53, p = 0.01]. The administration of RSP via NPs (both Prot1/RSP and Prot2/RSP) resulted in increased time in the center compared with all other treatments prior to amphetamine treatment (p ≤ 0.002; Cohen’s d ≥ 1.7) as well as after amphetamine treatment (p ≤ 0.04, Cohen’s d ≥ 1.39). A heat map analysis of the time occupied by the mice following the different treatments indicated that compared with saline (Fig. 7d_1, 2_), RSP (Fig. 7e_1, 2_), Prot.1 (Fig. 7f_1, 2_), and Prot.2 (Fig. 7g_1, 2_), both Prot.1/RSP (Fig. 7h_1, 2_) and Prot.2/RSP (Fig. 7i_1, 2_) groups exhibited significantly reduced activity in the open field arena.

These data support our hypothesis and demonstrate that RSP affects mouse behavior before and after amphetamine administration, with a more pronounced effect after amphetamine treatment. Moreover, the data show that the effects of RSP-encapsulated proteinoid NPs are more pronounced compared with free RSP. These differences are easily visible from the occupancy heat maps and plots produced by the ANY-maze software (Fig. 7d–i).

## Discussion

**Fig. 8:**
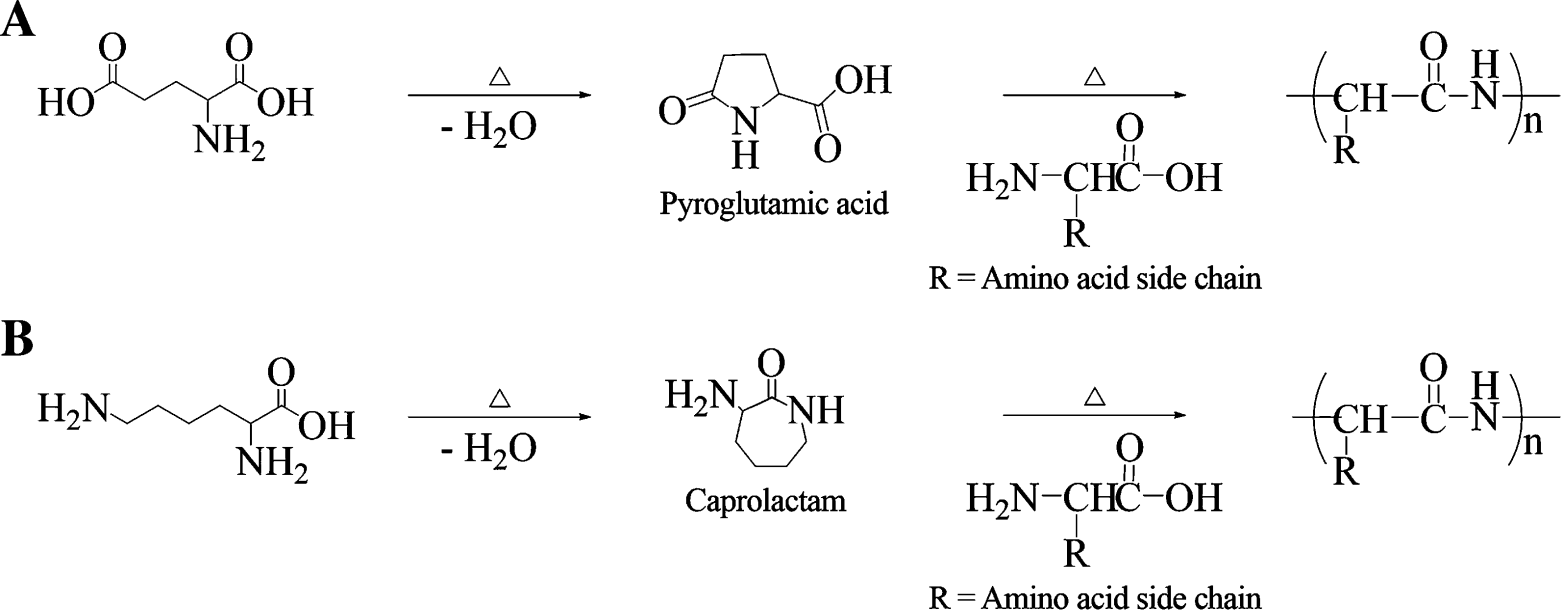
General scheme of proteinoid synthesis. Step-growth polymerization of amino acids through pyroglutamic acid (**a**) or caprolactam (**b**) to obtain acidic and basic proteinoids, respectively

Four different proteinoid polymers were synthesized by step-growth polymerization under anhydrous conditions and inert atmosphere [27, 54]. The proteinoids were synthesized from either Lys or Glu, along with Phe and His, with a weight ratio of 1:1:1 in absence or presence of 10% w/w PLLA, obtaining two lysine-based and two glutamic acid-based proteinoids (Prot.1–4) as shown in Table 4. Lysine and glutamic acid play a main role in the initiation process, as upon heating, these amino acids undergo homo-cyclization into pyroglutamic acid and caprolactam, respectively (Fig. 8) [55]. They serve as the solvent of the polymerization process and determine the basic and acidic nature of the final proteinoid [56–60]. The additional amino acids, phenylalanine and histidine, were selected for their ability to form π–π stacking, which enhances the rigidity of the proteinoid. The imidazole group of the histidine facilitates and expedites the proton sponge effect of endosomes, leading to endosomal/lysosomal escape [61]. PLLA was incorporated within the copolymer backbone to enhance biodegradability via ester functional groups.

**Table 4:** Composition of the different proteinoids

Proteinoid	l-Amino acid content	Weight ratio	PLLA (w%)
Prot.1	Lys, Phe, His	1:1:1	10
Prot.2	Glu, Phe, His	1:1:1	10
Prot.3	Lys, Phe, His	1:1:1	–
Prot.4	Glu, Phe, His	1:1:1	–

The total weight of the monomers content is 5 g

Table 1 exhibits the molecular weights and PDI values obtained for the proteinoids by GPC and the optical activity measured by polarimeter. Step-growth polymerization is a random process, hence it is unlikely to obtain high molecular weight with a narrow polydispersity index [62]. In the present study, however, the proteinoids resemble natural proteins, with Mw in the range of 132 to 168 KDa and low PDI (1.00–1.05) [34, 63]. The chirality of the proteinoids was not affected by the high temperatures used in the synthesis, and all proteinoids remain optically active after the procedure. This is significant and may be utilized in the design of stereospecific drug carriers [64].

Hollow and RSP-loaded proteinoid NPs were prepared, PEGylated, and evaluated in terms of diameter, SD, stability in aqueous continuous phase by ζ-potential, and DL, as shown in Table 2.

As expected, proteinoid NPs containing RSP have larger diameter compared to the corresponding hollow NPs. An increase in size is thus an indication that RSP is indeed entrapped within the proteinoid NPs.

The smaller diameters of Prot.1 and Prot.2 compared to Prot.3 and Prot.4 may result from the increased negative charge of the NPs that contain PLLA and the increased rigidity of hydrophobic segments induced by the higher content of phenylalanine and histidine in the interior part of these proteinoid particles.

It should be noted that both methods, DLS and cryo-TEM, measure the hydrodynamic diameter and distribution of the NPs, while SEM images measure the dry diameter. Figure 3, for example, illustrates SEM images of the Prot.1/RSP and Prot.2/RSP NPs, which yield significantly smaller dry diameters than the hydrodynamic diameters. Prot.1/RSP and Prot.2/RSP exhibit dry diameters of 74 ± 4 and 55 ± 5 nm, respectively, while the hydrodynamic diameters, as measured by DLS (Table 3) were 97 ± 2 and 86 ± 3 nm, respectively. DLS measurements take into consideration the hydrated water layers adsorbed onto the particle surface, which lead to increased diameter relative to the dry measurements.

The physical stability (against agglomeration) of the aqueous dispersions of both PEGylated hollow and RSP-loaded proteinoid NPs was studied by ζ-potential [65, 66]. The ζ-potential indicates the degree of repulsion between charged particles in the aqueous dispersion (negative or positive) and the ability to prevent particle aggregation. Table 2 presents the ζ-potentials at pH = 7.4 of the hollow and RSP-loaded proteinoid NPs. Prot.1 and Prot.2 are slightly more stable than Prot.3 and Prot.4, probably due to the added PLLA. The positive ζ-potential values for the basic proteinoid NPs (Prot.1,3) derive from the protonated amine groups on Lys and His, while for the acidic Prot.2,4 the values were negative due to carboxylate groups of Glu that are located on the outer shell of the particles, exposed to the aqueous continuous phase.

Table 3 exhibits the diameter and SD, ζ-potential and DL of the PEGylated Prot.1/RSP and Prot.2/RSP NPs loaded with increasing concentrations of RSP (10, 20, 30, 40 and 50% w/w). Table 3 illustrates that as the RSP concentration increases, the hydrodynamic diameter of the particles increases. Furthermore, the ζ-potential of the particles slightly increases with increasing RSP concentration, which is probably due to successful PEGylation process in which the free amino groups are conjugated to PEG.

Based on these results it can be concluded that PEGylated Prot.1 and Prot.2 NPs without and with 20% w/w RSP are the most suitable particles for continuation of the present study. Their physical stability as measured by ζ-potential and DL is higher than that of the other NPs, and they possess hydrodynamic diameters below 100 nm and thus have the potential to cross the BBB and target different regions of the brain and enhance the therapeutic effect of RSP.

In addition, leakage of RSP from Prot.1,2/RSP (20% w/w) aqueous dispersions (10 mg/mL) into the aqueous continuous phase was not detected after 3 months of storage at 4 °C, as described in the methods section. Long-term stability against agglomeration of the PEGylated Prot.1,2 and Prot.1,2/RSP (20% w/w) was accomplished by adding trehalose (10 mg) to the NP aqueous dispersions (1 mL containing 10 mg of NPs) followed by lyophilization. The NP powder was redispersed after 1 year in water to the original concentration. The measured diameter and SD of the redispersed NPs were the same as before lyophilization, confirming the long-term storage. Based on the results, as mentioned previously, the biological part of the study was conducted with Prot.1,2 (control) and Prot.1,2/RSP (20% w/w) NPs.

In vitro cell viability following 48 h treatment with Prot.1, Prot.2, and corresponding RSP-loaded NPs on cell proliferation of J774A.1 and Neuro-2α cells was determined by XTT, as illustrated in Fig. 4. Prot.1/RSP and Prot.2/RSP demonstrates no cytotoxicity on either cell line. These results indicate that RSP-loaded NPs do not affect the metabolic viability of the cells and are suitable candidates as drug carriers for antipsychotic drugs.

The NIR dye Cy7 was conjugated to the surface of the NPs in order to access cellular uptake by Neuro-2α cells. Figure 5A exhibits the intracellular fluorescence of Prot.1,2 and Prot.1,2/RSP obtained by FACS. The RSP-encapsulating proteinoid NPs exhibited a slightly higher fluorescence compared to their hollow counterparts.

Cells were also observed by a confocal fluorescent microscope, as shown in Fig. 5b. The images demonstrate that following 4 h of incubation, both Prot.1 and Prot.2 penetrate the cell and reach the cytoplasm. These results support the hypothesis that proteinoid NPs resemble proteins and can therefore be transported across the cell membrane in a similar manner.

In order to learn about the body distribution and blood half-life (Fig. 6), blood was drawn at different time points, and 4 h post injection the mice were sacrificed and organs were harvested. The half-life of both proteinoid NPs was calculated to be 20 min. In both cases, fluorescence was seen in internal organs related to the gastro-intestinal and urinal systems, which probably indicates that the NPs are eliminated from the body through the liver-intestine-colon and kidney-urine routes (Fig. 6b). Prot.2 was not seen in the colon 4 h post injection, possibly due to fast clearance of Prot.2 from the colon or the urinary tract. Interestingly, a specific fluorescent signal was detected in the brain, indicating that both proteinoids can cross the BBB.

When comparing the fluorescence in the brain to the fluorescence in all tested organs, ~ 1% of the total signal was found in the brain after 4 h. Figure 6c exhibits a notable difference between the fluorescent intensity of Prot.1 NPs compared to Prot.2 NPs in the brain tissue 4 h post injection. This presumably stems from the difference in number of conjugated Cy7 molecules on Prot.1 NPs prior to injection, which is also demonstrated at time zero in Fig. 6a. The results indicate that both Prot.1 and Prot.2 can potentially be used as drug carriers to transport therapeutics across the BBB and target the brain.

The results of the current study support our hypothesis that RSP loaded within a proteinoid NP delivery system can stabilize and increase the bioavailability of the drug and thereby enhance its activity. Our data clearly demonstrate that RSP encapsulated in proteinoid NPs can cross the BBB and act at its receptor targets. Moreover, the effects of RSP-loaded proteinoid NPs appear to be stronger than those of free RSP, suggesting higher efficacy at the receptor targets. A possible explanation for this difference is the NPs’ ability to penetrate into relevant brain areas and release the encapsulated RSP in the specific location. Furthermore, the encapsulated RSP is protected from decomposition by metabolic processes due to its entrapment within the proteinoid.

Nevertheless, several points should be addressed regarding these results. One interesting point is that RSP reduced activity both before and after amphetamine administration (Fig. 7). With the amphetamine-induced hyperactivity model, we expected that a tested compound at the appropriate dose would ameliorate amphetamine-induced activity but not baseline activity levels [67, 68]. However, it has long been noted that antipsychotic drugs induce sedation and therefore reduce activity levels in both humans [69] and animal models [70]. The induction of sedation in untreated versus amphetamine-treated mice is therefore dependent on dosage, whereby a higher dose can induce sedation and consequently reduce activity regardless of amphetamine treatment. It is possible that a detailed dose–response study can identify the dose of RSP in proteinoid NPs that will ameliorate amphetamine-induced hyperactivity with no effects on baseline locomotion. However, that was not the aim of this study in which the in vivo behavioral testing was designed merely as a proof of concept study for behavioral effects of RSP administered in a proteinoid NP drug delivery system compared with free RSP.

Beyond measures of activity, the results show that the time spent in the center of the open field arena was reduced by amphetamine but significantly increased by RSP in proteinoid NPs. Time in the center of an open field can be interpreted in the context of anxiety where procedures that increase anxiety result in lower time spent in the center, while procedures that reduce anxiety (such as administration of anxiolytics) increase time spent in the center [71]. The anxiogenic effects of amphetamine are well known [72] and therefore its effects to reduce center time in the current study are not surprising. However, the effect of encapsulated RSP to drastically increase time in the center of the open field may be interpreted in the context of anxiety but is more likely to be the result of the drastically lower locomotor activity, with possible freezing of some of the animals in the center of the open field. As animals in the test were placed in the center of the arena, it is possible that the long time in the center results from freezing and is unrelated to anxiety. It is indeed well known that high doses of antipsychotic drugs, including RSP, can induce catalepsy [73]. This is reinforced by the current study in which some of the animals spent over 1700 s (of an 1800 s session, before or after amphetamine treatment) in the center area with only minimal locomotor activity. The freezing response in animals prior to amphetamine treatment suggests caution in future translational studies as it is possible that relatively low doses will be enough to induce catalepsy. Yet, careful dose/response studies in humans should be sufficient to identify appropriate doses with stronger antipsychotic effects but without induction of catalepsy, as is the case for most antipsychotic treatments.

However, despite the limitations, it is clear that RSP encapsulated within proteinoid NPs and administered via peripheral injection reached the brain and induced strong and significant behavioral effects, as expected from a high dose of an antipsychotic drug. Therefore, it can be assumed that the encapsulated RSP is more effective than free risperidone.

## Conclusions

In this study, a new drug delivery system was investigated for the antipsychotic drug RSP. For this purpose, basic/acidic proteinoids were synthesized from l-Lys/Glu, l-Phe, l-His and PLLA via a step-growth polymerization process, yielding polymers with relatively high molecular weight and narrow size distribution. Hollow and RSP-loaded proteinoid NPs were prepared by self-assembly process, followed by PEGylation, and characterized for diameter and SD, ζ-potential and DL. Two types of NPs, Prot.1 and Prot.2, were chosen for further investigation based on diameter and stability and examined with various drug concentrations by HPLC, yielding optimal DL of 20% w/w.

The hollow and optimal RSP-loaded NPs were characterized by DLS, cryo-TEM and HR-SEM. The measurements confirmed that RSP was successfully encapsulated, resulting in nano-sized particles with diameters of 97 ± 4 and 86 ± 3 nm for Prot.1 and Prot.2, respectively. After long-term storage by freeze-drying in the presence of 1% w/v trehalose, particles were redispersed successfully to the original size.

In vitro studies of cytotoxicity and NP cell uptake by XTT, FACS and fluorescence microscopy demonstrated that both hollow and encapsulating NPs are non-toxic, penetrate the cell membrane, and reach the cytoplasm. In vivo biodistribution studies showed that Prot.1 and Prot.2 were distributed to all examined organs, including the brain after passing the BBB.

The antipsychotic effect of the RSP-loaded NPs was examined by behavioral testing using the amphetamine-induced hyperactivity screening model in mice. The results supported the hypothesis that the effect of RSP increases when entrapped within NPs. This may probably be explained by increased drug solubility and stability of the encapsulated RSP, leading to increased transportation across the BBB and better penetration to the specific area in the brain compared to the free drug. Hence, both Prot.1/RSP and Prot.2/RSP demonstrated excellent in vivo activity in reducing the psychotic behavior compared to free RSP, and can therefore potentially be used as an effective drug delivery system for RSP and other antipsychotic drugs. Future research envisages assessment of a wide range of behavioral tests and side effects of RSP-loaded proteinoid NPs at different concentrations. Ongoing studies of RSP-loaded proteinoids focus on optimal dosage and investigation of side effects for antipsychotic effect as well as for treatment of hyperactivity disorders.

## Methods

### Materials

The following analytical-grade chemicals were purchased from Sigma (Israel) in ≥ 98% purity and used without further purification: l-glutamic acid (Glu), l-lysine (Lys), l-phenylalanine (Phe), l-histidine (His), sodium hydroxide (NaOH), super-pure HPLC water, acetonitrile, trifluoroacetic acid (TFA), sodium chloride (NaCl), dimethyl sulfoxide (DMSO), trehalose, and Cyanine7-NHS (Cy7-NHS). Poly(l-lactic acid) (PLLA) MW 2 kDa was purchased from Polysciences (Warrington, PA, USA), methoxy polyethylene glycol succinimidyl succinimide (M-PEG-NHS, Mw 5 kDa) was obtained from JenKem Technology (Plano, TX, USA), and Risperidone was acquired from Teva Pharmaceutical Industries Ltd. (Kfar Saba, Israel). Water was purified by passing deionized water through an Elgastat Spectrum reverse osmosis system from Elga Ltd. (High Wycombe, UK). Dialysis membranes (1000 Da), Dulbecco’s modification of Eagle’s medium (DMEM), phosphate buffered saline (PBS), glutamine, penicillin/streptomycin, and XTT-based cell proliferation kit were purchased from Biological Industries (Bet Haemek, Israel). J774A.1 murine macrophages and Neuro-2α neuroblast cell lines were purchased from ATCC (Manassas, VA, USA). BALB/c and ICR (CD-1) mice were purchased from Harlan Biotech (Rehovot, Israel).

### Synthesis of proteinoids by thermal step-growth polymerization mechanism

Proteinoid synthesis was carried out as described in Table 4, in absence or presence of 2 kDa PLLA (10 w%) [63]. In brief, under N_2_ atmosphere, 5 g of Lys or Glu, Phe and His at 1:1:1 weight ratio was heated at either 140 or 180 °C, respectively. The mixture was stirred by a mechanical stirrer at 150 rpm for 45 min. The product was a highly viscous amber-brownish paste, which hardened upon cooling to room temperature. The residue was extracted by 30 mL of super-purified water, followed by lyophilization to yield dried proteinoid polymer powder.

### Proteinoid characterization

The molecular weights and polydispersity indices of the dried crude proteinoids were determined at 25 °C using GPC consisting of a Waters Spectra Series P100 isocratic HPLC pump with an ERMA ERC-7510 refractive index detector and a Rheodyne (Coatati, CA) injection valve with a 20 μL loop (Waters, MA). The samples were eluted with super-pure HPLC water through a linear BioSep SEC-s3000 column (Phenomenex) at a flow rate of 1 mL/min. The molecular weights of the proteinoids were determined relative to PEG standards (Polymer Standards Service-USA, Silver Spring, MD) with a Mw range of 100–450,000 Da, human serum albumin (HSA, 67 kDa) and bovine plasma fibrinogen (340 kDa) using Clarity chromatography software version 2.7.3.498 (DataApex, Prague, Czech Republic).

The optical activities of the proteinoids were determined with a PE 343 polarimeter (PerkinElmer). All measurements were performed in water at 589 nm and 25 °C.

### Preparation of hollow and RSP-loaded proteinoid NPs

Proteinoid NPs were prepared via self-assembly mechanism. Briefly, 100 mg of the dried proteinoid were added to 10 mL NaCl 10 µM aqueous solution. The mixture was heated to 80 °C and stirred at 250 rpm for 30 min until the crude proteinoid dissolved completely. Hollow proteinoid particles were formed as the mixture was left to cool to room temperature. RSP-loaded proteinoid particles were obtained in a similar procedure by the addition of RSP to the heated proteinoid solution. Due to the poor solubility of RSP in water, an appropriate concentration of RSP powder (0, 10, 20, 30, 40 and 50 weight% relative to the proteinoid) was first dissolved in DMSO (0.2 mL) and then heated to 80 °C. After both solutions reached 80 °C, RSP solution was added to the proteinoid mixture prior to the particle formation to achieve RSP-loaded proteinoid NP dispersion. Following preparation, the NPs were extensively dialyzed using a cellulose dialysis membrane with a molecular weight cut-off (MWCO) of 1000 Da against distilled water to remove the DMSO. The particle dispersion was filtered through a 3 μm glass microfiber membrane syringe filter (VWR EU, England) to remove excess RSP.

### PEGylation of hollow and RSP-loaded proteinoid NPs

PEGylated RSP-loaded proteinoid NPs (Prot./RSP) were prepared with M-PEG-NHS, Mw 5000 Da, which was reacted with the primary amine groups on the surface of the proteinoid NPs. Briefly, 100 μL of M-PEG-NHS dissolved in PBS (50 mg/mL) were added to RSP-loaded NPs (10 mg/mL) dispersed in sodium bicarbonate (2 mM, pH = 8) and the mixture was stirred at 150 rpm for 2 h at room temperature. The obtained PEGylated RSP-loaded NPs dispersions were then dialyzed through a cellulose membrane (1000 Da MWCO) against super-purified water to remove excess undesired materials. Similarly, PEGylation process was performed for the hollow proteinoid NPs.

### Preparation of NIR fluorescent PEGylated hollow and RSP-loaded proteinoid NPs

The NIR dye Cyanine7 NHS ester (Cy7-NHS) was conjugated to residual amine groups on the surface of the PEGylated hollow and RSP-loaded proteinoid NPs. Briefly, after coupling of the M-PEG-NHS, before dialysis, as described above, 50 μL of Cy7-NHS solution in DMSO (10 mg/mL) were added to the aqueous NP dispersion and stirred at 150 rpm for additional 2 h at room temperature. The NIR fluorescent conjugated NP dispersion was then extensively dialyzed through a cellulose membrane (1000 Da MWCO) against distilled water to remove the DMSO and excess Cy7.

### Proteinoid NP characterization

#### Diameter and size distribution

The hydrodynamic diameter and size distribution of the aqueous NP dispersions were measured at room temperature with a particle DLS analyzer model Nanophox (Sympatec GmbH, Germany). In addition, the diameter and size distribution of the NPs were measured with a cryogenic transmission electron microscope (cryo-TEM). Briefly, a small droplet of NP aqueous dispersion was placed on a perforated lacey carbon film supported on a TEM copper grid. The drop was blotted with a piece of filter paper, resulting in formation of thin films of 100–300 nm. The specimen was subsequently plunged into a reservoir of liquid ethane cooled by liquid nitrogen to ensure its vitrification (rapid freezing) and to prevent ice crystal formation. The vitrified specimen was transferred under liquid nitrogen and mounted on a cryogenic sample holder cooled to − 170 °C. All samples were observed under low-dose conditions. Vitrified samples were examined using a FEI T12 G2 Cryo-TEM operating at 120 kV and equipped with a Gatan 626 cryo-holder system. The mean diameter was determined by measuring at least 200 particles using the AnalySIS Auto image analysis software version 3.2 (Soft Imaging System GmbH, Germany).

Dried particle diameter and size distribution were measured with a scanning electron microscope (SEM, JEOL, JSM-840 Model, Japan). A drop of dilute particle dispersion in distilled water was spread on a glass surface and dried at room temperature. The dried sample was coated with carbon in vacuum before viewing under SEM. The average particle diameter and distribution were determined by measuring the diameter of more than 200 particles with the AnalySIS image analysis software.

#### ζ-Potential

The proteinoid NP surface potentials were measured in aqueous dispersion at pH = 7.4 at a concentration of 10 mg/mL using a Zetasizer 3000 HSa model ζ-potential analyzer (Malvern Instruments Company, England).

#### HPLC

HPLC analysis of the RSP drug loading (DL) was carried out by a Spectra System HPLC equipped with a UV/vis detector (Thermo Scientific, USA) and a reverse phase C18 column (75 mm × 4.6 mm, Phenomenex, USA). The mobile phase was water and acetonitrile, both containing 0.1% aqueous TFA solution, at a flow rate of 1 mL/min at 25 °C wavelength was set at 285 nm [74]. Calibration standard solutions were prepared and used by diluting an appropriate volume of stock standard solution in ethanol, yielding concentrations of RSP in the range of 60–200 μM. RSP-loaded NPs were diluted by ethanol and sonicated in an ice-water bath for 20 min prior to injection. The sonication and the added ethanol cause the proteinoid NPs to disassemble and elute the RSP. The injection volume was set to 10 μL for all standard samples in the range of 60–200 μM RSP. The weight of drug in each sample was calculated using the calibration curve.

#### Leakage of RSP

A dialysis technique was applied to study RSP leakage from the Prot./RSP NPs [7]. Briefly, RSP (20% w/w) loaded proteinoid aqueous dispersions (10 mg/mL) were kept in the refrigerator at 4 °C for 3 months followed by extensive dialysis against water. The diameter and size distribution of the RSP-loaded proteinoid NP aqueous dispersions and their RSP content were measured by DLS and HPLC, respectively.

#### Proteinoid NPs long term storage stability

Long-term storage of the Prot./RSP NPs was investigated by freeze-drying methodology. Briefly, 10 mg of trehalose were added to 1 mL of NP aqueous dispersion (10 mg/mL), followed by lyophilization to dryness and storage at 4 °C. The NP powder was redispersed in water to the original concentration (in 1 mL) and the diameter and size distribution were determined by DLS.

### Cell viability analysis for proteinoid NPs

In vitro toxicity of PEGylated hollow and RSP-loaded NPs was tested using the XTT (2,3-bis-(2-methoxy-4-nitro-5-sulfophenyl)-2H-tetrazolium-5-carboxanilide-salt) assay in two murine cell lines: J774A.1 macrophage and Neuro-2α neuroblast cells. The cells were seeded in a 96-well plate at a density of 1 × 10^4^ cells/well in 100 μL culture medium and grown in a humidified 5% CO_2_ atmosphere at 37 °C. The PEGylated hollow and RSP-loaded NPs were freshly dispersed in water containing 1% DMSO aqueous solution, and NPs dispersions were added to 95% confluent cell culture in culture medium.

Cells were treated with PEGylated hollow and RSP-loaded NPs at a concentration of 0.5 mg/mL with RSP concentration of 10 µg/mL. Cells treated with an equivalent amount of free RSP and untreated cells served as control groups. The cell cultures were further incubated at 37 °C in a humidified 5% CO_2_ incubator and examined for cellular viability after 48 h. The cell viability was calculated as shown in the manufacturer’s protocol of the XTT kit, and the experiment was repeated twice. All samples were tested in six-fold and analyzed by UV spectrophotometer at 492 nm with reference wavelength of 620 nm. The assay assesses the metabolic rate of the mitochondria in the cells following NP exposure.

### Proteinoid NP cell uptake

The ability of proteinoid NPs to penetrate the cell membrane was studied using flow cytometry. FACS of Cy7-conjugated NPs within the cells was evaluated by a FACS Aria III (BD) cell sorter. Cells were treated with Cy7-conjugated PEGylated hollow and RSP-loaded Prot.1 and Prot. 2 NPs (0.5 mg/mL) for 24 h at 37 °C prior to analysis. After incubation, the cells were washed with fresh medium and stained with Hoechst 33342 (nucleus staining, 1 μg/mL). In order to maximize cell viability and minimize mechanical perturbations, the flow rate was set to 1.1 (minimum). For Cy7 analysis, a 633 nm excitation laser was used with a filter. The data was processed by FlowJo v7.6.4. Live cell imaging was performed on an Olympus FV-1000 confocal microscope.

For the Cy7-conjugated particles, 50,000 Neuro-2α cells were grown on 15 mm glass coverslips pretreated with 0.1% gelatin for 48 h Cells and treated with Cy7-conjugated PEGylated hollow NPs and Prot. 1,2/RSP NPs (0.1 mg/mL) for 24 h at 37 °C. After incubation, cells were washed with PBS, fixed by cold (− 20 °C) methanol for 20 min and stained with DAPI (nucleus staining, Fluoroshield, Sigma). Cell imaging was performed on the Olympus confocal microscope.

### In vivo biodistribution studies

Male BALB/C mice (n = 12, Harlan Laboratories, Israel) were utilized under a protocol approved by the Institutional Animal Care and Use Committee at Bar-Ilan University. The biodistribution of the Cy7 fluorescent NPs was studied in normal 8-week-old mice weighing 25–30 g at the time of experiment.

For this purpose, 100 μL of the Cy7-conjugated PEGylated hollow proteinoid and Prot./RSP NPs and PBS dispersion (0.01 mg/kg body weight) were administered to the mice via IV injection to the tail vein at a concentration of 0.3 mg/kg. Blood samples were taken immediately after NP injection at 0, 30 min, 1 h and 4 h, mice were euthanized by CO_2_ 4 h post injection, and organs were harvested for imaging (brain, colon, heart, lungs, liver, spleen, kidneys, duodenum and blood). Each group consisted of five mice, and two nontreated mice served as negative control.

Fluorescence images were acquired using a Maestro II in vivo fluorescence imaging system (Cambridge Research & Instrumentation, Inc., Woburn, MA) The system is equipped with a fiber-delivered 300 W xenon excitation lamp, and images can be acquired from λ = 500–950 nm by a 1.3 megapixel CCD camera (Sony ICX285 CCD chip). A deep red excitation/emission filter set was used for our experiments (λex: 700–770 nm, λem > 780 nm). The liquid crystal tunable filter was programmed to acquire image cubes from λ = 780 to 840 nm with an increment of 10 nm per image. The camera was set to exposure times of 20,000 ms (brain), 400 ms (colon), 2000 ms (heart), 1000 ms (lungs), 3000 ms (liver), 20,000 ms (spleen), 3000 ms (kidneys), 3000 ms (duodenum), and 5000 ms (blood). Fluorescence intensity measurements were performed using ImageJ NIH (National Institutes of Health) software version 1.48v.

### In vivo psychopharmacological studies

An amphetamine-induced hyperactivity test was used to examine the possible effects of PEGylated hollow proteinoid and Prot.1,2/RSP NP dispersions at the behavioral level. Subjects were male ICR (CD-1^®^) outbred 8-week-old mice (n = 48) weighing 25–30 g at the time of experiment. Mice were treated with either saline (control), free RSP, or NP dispersion. Specifically, 100 μL of either NP dispersion at a concentration of 2 mg/mL or a solution of 10 mg RSP dissolved previously with 1% (v/v) DMSO in 5 mL super-purified water, giving a final concentration of 2 mg/mL, and saline were injected IV via the tail to the mice, immediately before the start of the behavioral test according to the t_1/2_ of the NP dispersion in the blood. Following treatment, each mouse was placed in the center of a 40 × 40 cm open field arena with 30 cm high walls where activity was monitored using the ANY-maze automated video tracking system (Stoelting, IL, United States) for a 60 min session. Thirty min after the start of the session, mice were briefly taken out of the arena, injected IP with amphetamine dissolved in saline at a 5.0 mg/kg dose and 10 mL/kg volume, and immediately placed back in the arena for additional 30 min. This is a within subject design for the effects of amphetamine and across subject design for the effects of RSP. The experiment was repeated twice.

### Statistical analysis

Data for the behavioral experiment were analyzed using Statistica 13.0 (Dell, Texas) software. Analysis included a mixed ANOVA with RSP treatment as a main factor and amphetamine administration as a repeated measures factor (pre and post amphetamine injection). Significant effects were followed by Scheffe post hoc tests. Effect sizes (Cohen’s d) were calculated using an online calculator (https://www.uccs.edu/lbecker/). One mouse from group Prot.1/RSP was excluded from the statistical analysis as an outlier (more than two standard deviations away from the group mean).

### Ethical approval

All experiments complied with the ARRIVE guidelines (Animal Research: Reporting of In Vivo Experiments) and were carried out in accordance with the National Institute of Health and the Israeli Ministry of Health Guide for the Care and Use of Laboratory Animals (NIH Publications No. 80-23). All procedures were approved by the Bar-Ilan University Institutional Animal Care and Use Committee (IACUC).

## Data Availability

All data generated or analyzed during this study are included in this published article.

## Abbreviations

ANOVA: Analysis of variance
Cryo-TEM: Cryogenic transmission electron microscopy
DL: Drug loading
DLS: Dynamic light scattering
GPC: Gel permeation chromatography
HPLC: High performance liquid chromatography
IP: Intraperitoneal
IV: Intravenous
M-PEG-NHS: Methoxy polyethylene glycol succinimidyl succinimide
NPs: Nanoparticles
NIR: Near infrared
PDI: Polydispersity index
PLLA: Poly(l-lactic) acid
Prot./RSP: Risperidone-loaded proteinoid
RSP: Risperidone
SEM: Scanning electron microscope
SD: Size distribution
XTT: 2,3-Bis-(2-methoxy-4-nitro-5-sulfophenyl)-2H-tetrazolium-5-carboxanilide
